# Lower-limb joint-coordination and coordination variability during lateral shuffle in colleague students with different vision acuity

**DOI:** 10.1038/s41598-026-40892-x

**Published:** 2026-02-21

**Authors:** Huihui Wang, Xiaonan Wu, Lin Zhang, Huali Sun, Aochuan Xue

**Affiliations:** 1 School of Sports and Health, Zunyi Medical University, Zunyi, Guizhou China; 2 School of Physical Education and Health, East China Normal University, Shanghai, China

**Keywords:** Visual intervention, Lateral shuffle, Movement analysis, Joint coordination, Health care, Risk factors

## Abstract

Vision serves as a crucial determinant influencing human posture control. However, there is a paucity of research quantifying the relationship between vision intervention and motion control. The lateral shuffle is a prevalent movement pattern in sports and a representative movement associated with sports injuries. This study systematically evaluated the effects of various visual conditions (normal vision, + 150° convex lens, and + 450° convex lens) on bilateral lower limb coordination and variability at the hip-knee and knee-ankle joints during lateral shuffle movements. This study recruited 29 male college students with normal vision, with 19 participants meeting inclusion criteria (average age 19.84 ± 0.83 years, height 176.74 ± 5.55 cm, weight 68.51 ± 12.10 kg, BMI 21.84 ± 2.56 kg/m^2^). Kinematic data were collected using the PN3 Pro device under various visual conditions, which were then used to compute lower-limb coordination and coordination variability via the continuous relative phase method.Data analysis was performed using SPSS 24 software, with one-way repeated measures ANOVA employed to evaluate the impact of myopia severity on coordination, with effect sizes measured by partial eta squared (ηp^2^). Visual interventions significantly impacted lower limb coordination, demonstrating pronounced joint function gradient effects and lateral asymmetry. Compared to hip-knee coordination, knee-ankle coordination exhibited greater sensitivity to visual interference, with effect sizes ranging from moderate to large (ηp^2^, 0.129–0.418). Particularly under + 450° convex lens visual conditions, the effect size (ηp^2^) of Left knee-ankle coordination reached 0.418, explaining up to 41.8% of variance (*p* < 0.05). +450° convex lens conditions significantly increased coordination variability, supporting a dose-response relationship, with knee-ankle coordination showing heightened sensitivity. Interventions for varying degrees of myopia can impact lower limb postural control during lateral movements. The alterations in lower limb coordination predominantly affect the knee and ankle joints, rendering these two areas particularly susceptible to injury.

Postural control is the foundation of human daily activities and motor skills. It relies on the integration of multisensory information from visual, vestibular, and somatosensory systems, as well as the coordinated ability of lower limb joints^1^. Among these, vision plays a critical role in environmental perception, disturbance anticipation, and the maintenance of bodily stability^2^. Previous studies have primarily focused on static balance control in individuals under complete visual masking^2^. However, in daily life, the human body is mostly in a state of motion, and the challenges of postural control during dynamic activities are far more complex and difficult than those in static standing. Currently, it remains unclear how individuals with visual interference adjust lower-limb coordination and form specific compensatory strategies to maintain stability during dynamic tasks, as well as the underlying neuromuscular control mechanisms involved. The lateral shuffling task, which requires rapid lateral movement and weight transfer, is of particular interest. The mechanisms by which visual influences movement patterns in such tasks urgently require in-depth investigation. Elucidating this issue will not only deepen theoretical understanding of sensorimotor compensation mechanisms but can also provide crucial scientific evidence for developing targeted fall-prevention strategies and rehabilitation interventions.

Myopia, as a common ophthalmic disorder, can affect the biomechanical performance of college students during exercise, leading to misjudgment in movement, alterations in coordination, and ultimately resulting in injuries^3^. In addition, the connection to injury prevention remains scientifically unresolved. Undertaking quantitative study on the elements affecting vision in athletic activities can provide a theoretical basis for the prevention of sports-related injuries and help develop strategies to mitigate the impact of myopia on sports performance.

The lateral shuffle is a common movement pattern in sports. Studies have shown that soccer players use the lateral shuffle for 3.9 to 4.5% of the total match time, while basketball players utilize it for 18.1 to 42.1% of the match^4^. In a single basketball game, the lateral shuffle can occur up to 300 times^5^, highlighting its importance in sports. During the lateral shuffle, subjects are required to adapt to different situations on the field by quickly and effectively shifting their center of gravity. This movement, which is frequent and demanding, necessitates high precision in visual judgment^6,7^ and coordinated bodily control^8^, otherwise, it may increase the risk of injury and impair athletic performance^9^.

Current research on biomechanical factors of the lateral shuffle primarily focuses on kinematics, kinetics, balance ability, and proprioception^5–11^. Motor coordination refers to the nervous system’s ability to systematically organize multi-joint and muscle groups in spatiotemporal dimensions to efficiently and precisely execute predetermined movements^12^. Its core lies in the central nervous system integrating multisensory information to resolve the “degrees of freedom” issue in human motion, thereby forming stable and controllable functional movement patterns^12^. In movement, coordination serves as the foundation for all complex motor skills, determining the efficiency of action learning and the fluidity and precision of final performance^13^. Furthermore, coordination significantly enhances movement economy by optimizing muscle force sequence and joint coordination^14^. When performing tasks requiring rapid judgment and response, such as lateral shuffle steps, athletes rely heavily on accurate visual information extraction^15^. Thus, investigating the impact of visual interference on lower limb coordination patterns and their variability can directly reveal the intrinsic mechanisms of sensory-motor integration, providing a critical theoretical perspective for understanding motor control deficits and developing targeted training or rehabilitation strategies.

This study hypothesizes that during the lateral shuffle step performed by college students, varying degrees of visual interference (using + 150° and + 450° convex lenses) will systematically disrupt inter-joint coordination patterns in the lower limbs, specifically manifesting as reduced coordinated continuity and increased coordination variability.

## Subjects and methods

### Subjects

A priori power analysis showed that a minimum sample size of 24 participants (G*Power, Version 3.1, University of Dusseldorf, Germany) was needed to detect a moderate effect size for One-way repeated-measures ANOVA, with the effect size of 0.50, α error of 0.05, and power error of 0.90^16,17^. 29 male college students with normal vision were randomly selected as research participants at Zunyi Medical University. After applying the predefined exclusion and inclusion criteria, a total of 19 subjects who met the experimental requirements were ultimately selected. The average age of subjects was 19.84 ± 0.83 years, the average height was 176.74 ± 5.55 cm, the average weight was 68.51 ± 12.10 kg, and the average BMI was 21.84 ± 2.56 kg/m^2^. Informed consent was obtained from all subjects. This study has been approved by the Medical Ethics Committee of Zunyi Medical University (2022, No.2–014). Inclusion Criteria: Bilateral visual acuity greater than 1.0; no joint injuries in the past six months, with normal muscle strength and joint range of motion; good physical health and psychological well-being; no bad exercise habits, with normal neurological function and physical development; age range between 18 and 25 years old; regular engagement in sports activities (e.g., basketball, football, tennis) with established good athletic habits; subjects voluntarily participated in the study after being informed of the test procedures in advance. Exclusion Criteria: poor vision, with visual acuity of less than 1.0 in either eye, Recent history of joint injuries or other sports-related injuries, Regular exercise training was carried out within three months. prior relevant movement training within three months before participating in the experiment.

### Myopia data collection

The binocular vision and unilateral vision of the subjects were randomly tested, and the three conditions of normal vision, wearing a 150°convex lens, and wearing a 450°convex lens were also randomly selected. Three tests were conducted under each condition, and the specific testing conditions and requirements have been described in previous literature^3,18^. Visual simulation glasses were designed by the experimental team and processed by Zunyi Guangming Optical Equipment Factory. All methods were performed in accordance with the relevant guidelines and regulations.

## Methods

### Kinematic data collection

**Fig. 1:**
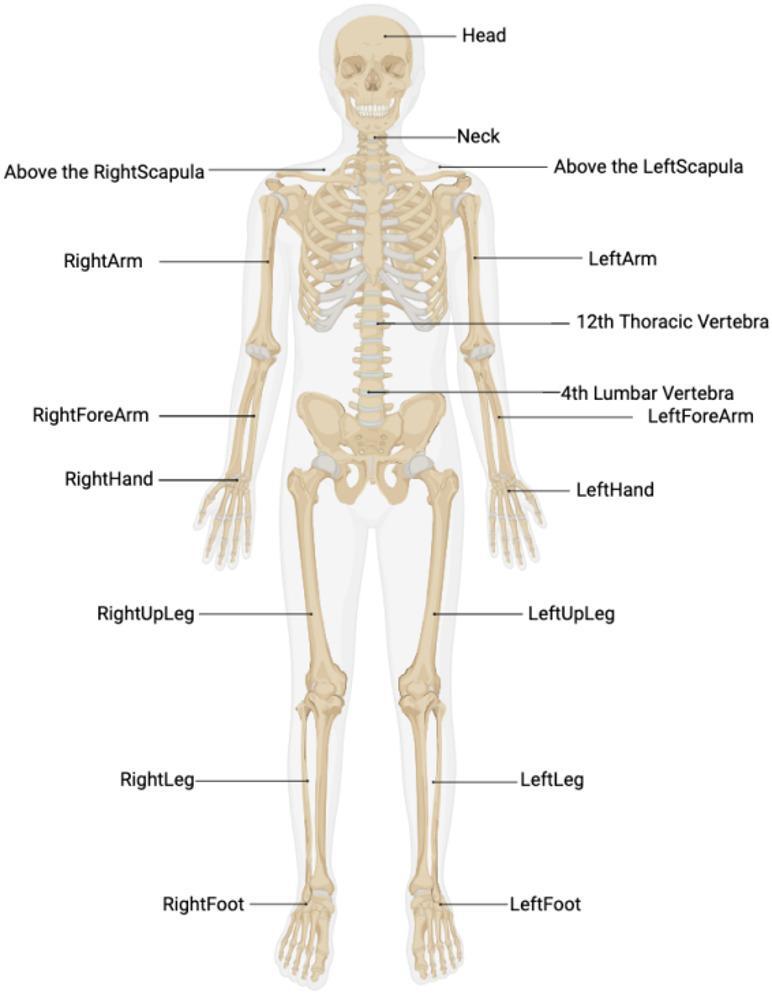
The model of inertial sensor position.

Kinematic parameters of the lateral shuffle were captured using the PN3 Pro system (Perception Neuron 3 Pro) with a sampling rate of 60 Hz. Prior to the tests, morphological data of the subjects were collected to build a personalized model. This data included height, weight, arm span, head height, neck length, shoulder width, trunk length, thigh width, upper arm length, forearm length, hand length, thigh length, calf length, foot length, and heel height. Import the subjects’ measured morphological data into the software to establish a personalized model. Then, 17 wireless nine-axis inertial sensors were fixed in the corresponding locations of the subject: the midpoint of the forehead, thoracic vertebrae, lumbar vertebrae, as well as the scapula, upper arm, forearm, thigh, lower leg, instep, and back of the hand on both sides (Fig. 1).

Kinematic data were collected for standing calibration and lateral shuffle trials (1.5 ms^−1^ ± 0.02ms^−1^). A staff member instructed participants on exercise precautions using standardized language. Prior to testing, participants were allowed to freely move and practice designated movements within a 10 × 3 m rectangular wooden walkway. Participants waited at the starting position and began moving toward the endpoint upon hearing the “start” command. During testing, lateral shuffle movements must follow these specifications: stand with feet parallel, knees slightly bent, torso leaning forward, and arms extended laterally; when shuffling leftward, step leftward with the left foot while simultaneously pushing off the ground with the right foot to slide it toward the left foot, maintaining a consistent distance between feet throughout the movement, with step length determined by natural stride. The subjects completed 3 lateral shuffle trials at the required speed under each visual condition^11^. For all lateral shuffle trials, subjects were instructed to keep their hands at the side of the body and approximately at their hip height^19^.

### Data processing

Data were further reduced to one lateral shuffle cycle, allowing for data to be interpolated 100% of the completed lateral shuffle cycle (101 data points) using a cubic spline function. Inter-joint coordination was quantified by the Continuous relative phase (CRP) technique, a measure with high reliability and validity for calculating coordination between two segments or joints by examining the difference of phase planes^20^. Initially, phase planes were constructed as a function of a segment’s angular position versus the angular velocity^21^. Each segmental angular position of each data point in the sagittal plane was normalized to a range between 1 and − 1 with zero located on its midpoint and angular velocity was normalized to the maximum absolute velocity during the movement^22,23^. Then, the phase angles were calculated for each data point through the entire lateral shuffle cycle using the arctangent function^23^. Next, the CRP was calculated by using the absolute value of the difference between the proximal and distal segment phase angles, and the CRP curves were determined from the mean of three trials of each subject and then calculating the average of mean curve across all subjects^23^. The CRP values were restricted between 0°and 180°. CRP values close to 0°represented “in-phase”, which denoted that the two segments move in the same direction at the same time, and CRP values close to 180°represented “anti-phase”, which denoted that the two segments move in the opposite direction at the same time^24^.

The indicators for quantifying the amplitude and variability of the CRP curve were the mean absolute relative phase (MARP) and deviation phase (DP)^23,25^. Lower MARP values represented the two coupling segments more “in-phase”, and higher values indicated more “anti-phase”. Lower DP values represented less variability or less instability between the two segments. A lateral shuffle cycle is defined as the interval from the left foot’s initial ground contact to its subsequent ground contact. The cycle of lateral shuffle was divided into four distinct periods for the left leg and right leg, as detailed in Table 1. MARP and DP values were calculated for one cycle and the four phases in both segment pairs (hip-knee, knee-ankle) of bilateral lower limbs in the sagittal plane.

**Table 1:** Summary of Abbreviations.

Acronym	Full name	Explanation
LDSP	Double support phase for the left double support phase	From the moment the left foot touches the ground to the moment the right foot leaves the ground
RDSP	Double support phase for the right double support phase	
LLSP	Left leg support phase	From the moment the right foot leaves the ground to the moment the left foot leaves the ground
RSP	Right swing phase	
LFP	Flight phase	From the moment the left foot leaves the ground to the moment the right foot touches the ground
RFP	Flight phase	
RLSP	Right leg support phase	From the moment the right foot touches the ground to the moment the left foot touches the ground
LSP	Left swing phase	
LOC	The entire cycle for Left leg	A lateral shuffle cycle is defined as the interval from the left foot’s initial ground contact to its subsequent ground contact.
ROC	The entire cycle for Left leg	
RHK	Right hip-knee	
LHK	Left hip-knee	
RKA	Right knee-ankle	
LKA	Left knee-ankle	

### Statistical analysis

The obtained data were analyzed statistically using SPSS, version 24. Shapiro-Wilk test was used to check for the normal distribution of data. The impact of varying degrees of myopia on the coordination of lateral shuffle was analyzed using one-way repeated measures ANOVA, Mauchly’s Test of Sphericity was used to verify whether the covariance matrix among repeated measurement variables meets the “Sphericity” condition. Post hoc testing was conducted using the Least Significant Difference (LSD) method for multiple comparisons, with the significance level was set at α = 0.05. Effect sizes (ES) were used to evaluate the clinical importance of mean difference.η_p_^2^(Partial Eta Squared), a measure of effect size, quantifies the reliability of lower limb coordination under the sole influence of visual conditions. 0.01 ≤ η_p_^2^ < 0.06 small effect, 0.06 ≤ η_p_^2^ < 0.14 Medium Effect,η_p_^2^ ≥ 0.14 Large Effect^26^.

## Results

### Inter-segment coordination amplitude(MARP)

**Fig. 2:**
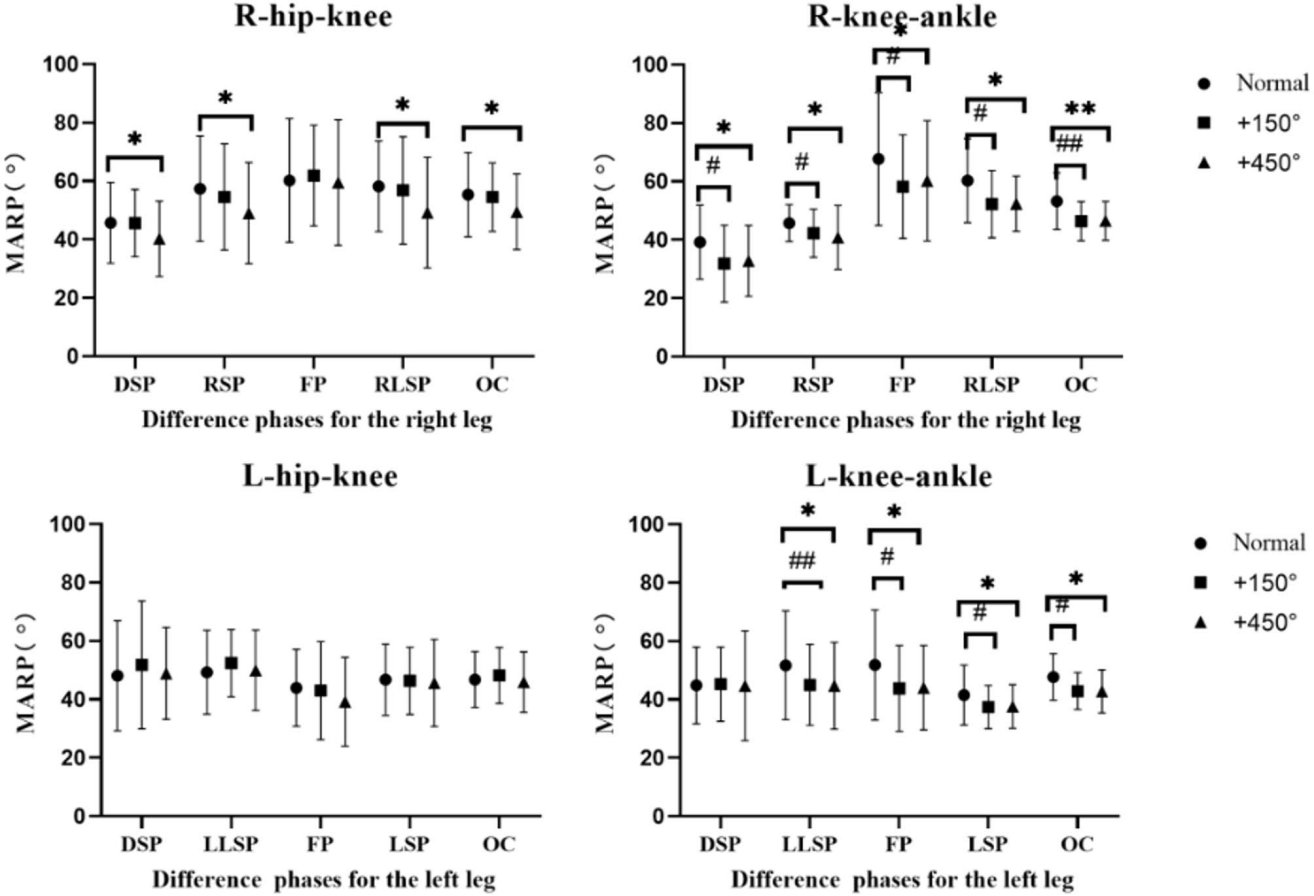
Mean absolute relative phase(MARP)for RHK, RKA, LHK, LKA coordination was presented in different phases.RHK: right hip-knee, RKA: right knee-ankle, LHK: left hip-knee, LKA: left knee ankle, DSP: double support phase, LLSP: left leg support phase, FP: flight phase, LSP: left leg swing phase, OC: one cycle of lateral shuffle, RLSP: right leg support phase, RSP: right leg swing phase. A symbol of * represents statistical significance of *p*<0.05 between normal vision and with + 450°convex lens, and two symbols represents statistical significance of *p*<0.01; a symbol of # indicates statistical significance of *p*<0.05 between normal vision and with + 150°convex lens for the respective lateral shuffle.

As shown in Table 2; Fig. 2, significant differences were observed between normal vision and conditions with + 450° convex lens conditions in various phases of the right hip-knee (RHK) coordination during the lateral shuffle step cycle, including the right double support phase (RDSP), right swing phase (RSP), right leg support phase (RLSP), and the entire cycle (OC). The effect size was moderately large, with partial eta-squared values (η_p_^2^) ranging from 0.113 to 0.125 (*p* > 0.05), indicating that visual interventions explained approximately 11.3% to 12.5% of the coordination variability, though not reaching statistical significance.

In comparison, the coordination of the mean absolute relative phase (MARP) between the knee and ankle joints exhibited more extensive and significant impacts. For right knee-ankle (RKA) MARP, all three visual conditions demonstrated significant main effects across all four phases and the entire cycle. The effect sizes ranged from moderate to large, with partial eta-squared (η_p_^2^) values between 0.129 and 0.223. Specifically, the right leg support phase (RLSP, η_p_^2^_=_ 0.152, *p* = 0.031) and the entire cycle (OC, η_p_^2^= 0.223, *p* = 0.005) showed statistically significant results, indicating that visual interventions could explain up to 22.3% of the variance.

Similarly, a significant main effect was observed in left knee-ankle (LKA) MARP, with differences primarily evident during the left leg support phase (LLSP), flight phase (FP), left swing phase (LSP), and the entire cycle (OC). The effect demonstrated high intensity and statistical significance, with partial eta-squared (η_p_^2^) values ranging from 0.158 to 0.418 (*p* < 0.05). Particularly strong effects were observed in the left leg support phase (LLSP, η_p_^2^ = 0.170, *p* = 0.020) and the entire cycle (OC, η_p_^2^= 0.418, *p* = 0.004), indicating that visual conditions explain up to 41.8% of the variation in left knee-ankle MARP.

**Table 2:** Partial eta squared in tests of within-subjects effects on MARP (η_p_^2^, P).

Left hip-knee	LDSP	LLSP	LFP	LSP	LOC
	0.031, 0.521	0.075, 0.206	0.004, 0.769	0.011, 0.630	0.073, 0.471
Left knee-ankle	0.001, 0.984	0.170, **0.020**	0.287, 0.034	0.158, 0.027	0.418, 0.004
	RDSP	RSP	RFP	RLSP	ROC
Right hip-knee	0.121, 0.067	0.113, 0.081	0.005, 0.891	0.125, 0.060	0.125, 0.060
Right knee-ankle	0.151, 0.032	0.129, 0.055	0.129, 0.055	0.152, 0.031	0.223, 0.005

### Inter-segment coordination variability (DP)

This study investigates how various visual conditions (normal vision, + 150° convex lens, and + 450° convex lens) affect the coordination and variability in inter-joint coordination of the bilateral lower limbs during lateral shuffle movements and presents the corresponding effect sizes (η_p_^2^) and p-values.

**Fig. 3:**
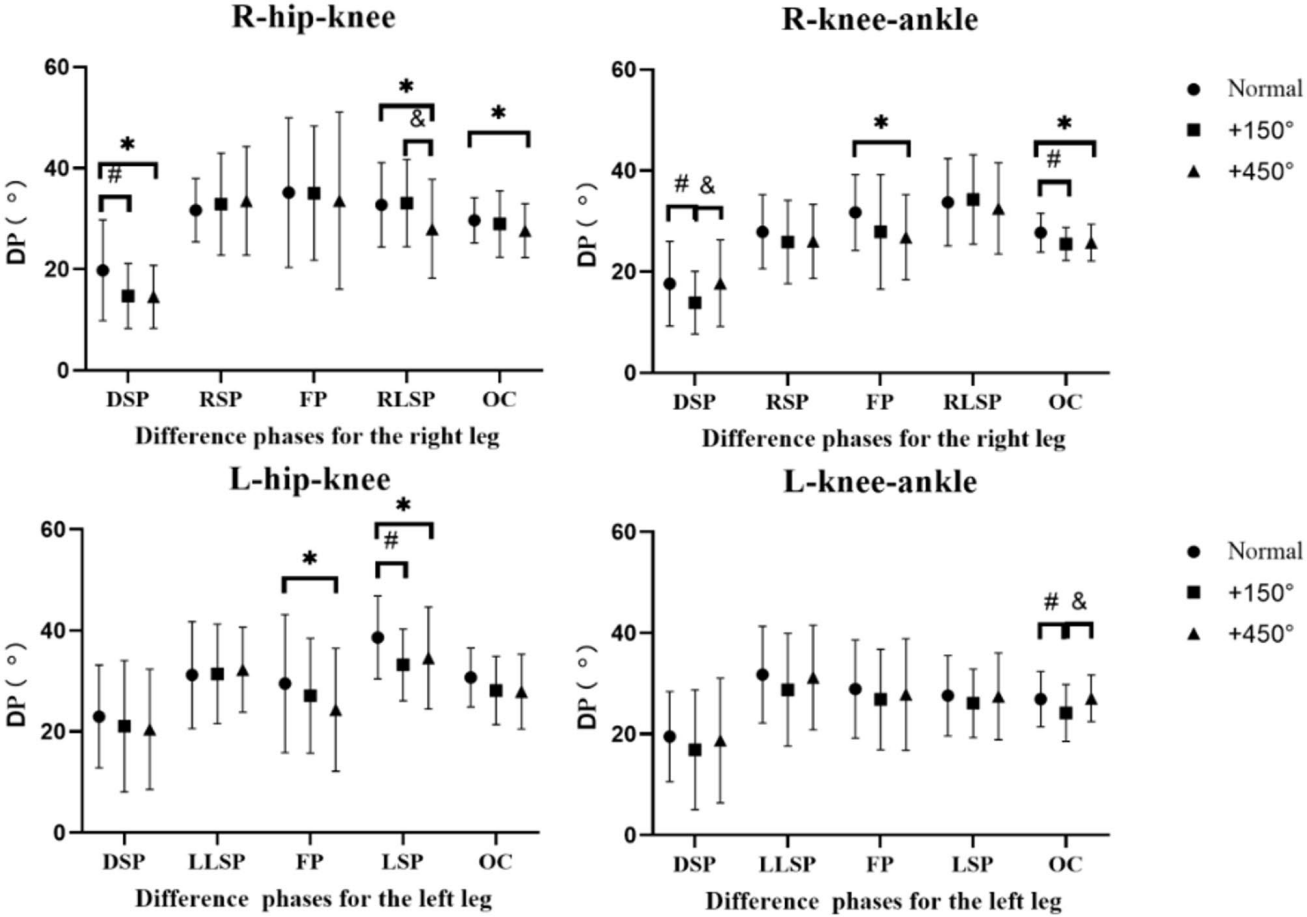
Deviation Phase (DP) for RHK, RKA, LHK, LKA coordination variability was presented in different phases. A symbol of & represents statistical significance of *p*<0.05 between with + 150°convex lens and with + 450°convex lens.

As shown in Table 3; Fig. 3, Under different visual conditions, the hip-knee coordination variability (DP) in the left lower limb did not show any significant differences across various phases of the lateral shuffle(*p* > 0.05), with small effect sizes (η_p_^2^ ranging from 0.004 to 0.134). Similarly, knee-ankle DP demonstrated no significant differences at any stage (*p* > 0.05), with small effect sizes (η_p_^2^ ranging from 0.018 to 0.124). Although the p-value for hip-knee DP during the left foot swing phase (LSP) approached significance (*p* = 0.049, η_p_^2^ = 0.134), it did not reach the statistical threshold.

The test results for the right lower limb revealed significant differences in DP across specific phases. Hip-knee DP showed significant inter-group differences during both the right leg double support phase (RDSP) and right leg support phase (RLSP) (*p* = 0.020, η_p_^2^ = 0.170; *p* = 0.008, η_p_^2^ = 0.204), indicating that blurred vision significantly affects right hip-knee coordination control. The knee-ankle coordination showed significant differences across the entire right foot cycle (ROC) (*p* = 0.037, η_p_^2^ = 0.145), while other phases such as the right leg swing phase (RSP) and the flight phase (RFP) showed no significant differences (*p* > 0.05).

**Table 3:** Partial eta squared in tests of within-subjects effects on DP (η_p_^2^, P).

left hip-knee	LDSP	LLSP	LFP	LSP	LOC
	0.027, 0.564	0.004, 0.918	0.088, 0.145	0.134, 0.049	0.053, 0.319
Left knee-ankle	0.030, 0.523	0.033, 0.499	0.018, 0.678	0.021, 0.635	0.124, 0.062
	RDSP	RSP	RFP	RLSP	ROC
Right hip-knee	0.170, **0.020**	0.041, 0.418	0.011, 0.796	0.204, **0.008**	0.044, 0.391
Right knee-ankle	0.114, 0.079	0.046, 0.374	0.085, 0.155	0.012, 0.782	0.145, 0.037

## Discussion

This study examined the alterations in lower-limb joint coordination amplitude (MARP) and variability (DP) among college students with normal vision, as well as those wearing + 150° convex lens and + 450° convex lenses during lateral shuffle.

## Inter-segment coordination amplitude (MARP)

This study systematically investigated how varying degrees of visual acuity affect lower limb joint coordination during lateral shuffle movements. The findings revealed that changes in visual precision significantly impair lower limb motor control, exhibiting distinct “distal specificity” and “left-side (landing legs) dominance” characteristics. Key results indicate that knee-ankle coordination was more sensitive to visual interference, particularly in the left limb and under high-intensity conditions (+ 450° convex lens). These findings reveal the mechanism of visual influence on coordination amplitude and coordination variation during lateral shuffle, providing a basis for sports training guidance and injury prevention.

First, this study observed that knee-ankle coordination demonstrated significantly stronger responses to visual interference compared to hip-knee coordination, indicating a clear joint function gradient effect of visual feedback in motor control. This finding aligns with the “distal priority” theory in motor control, which suggests that during lateral movements requiring rapid postural adjustments, the body relies more on visual information to precisely regulate distal joints (e.g., ankles) for maintaining dynamic stability^27^. Additionally, coordination variability increased markedly under + 450° convex lens conditions, further confirming a dose-response relationship between visual distortion severity and motor output errors. This finding is consistent with previous reports in gait and balance control studies^28^.

It is noteworthy that although significant differences in right hip-knee coordination were observed under varying degrees of visual intervention conditions during certain phases of the lateral shuffle, the effect size remained significantly lower than the threshold.This indicates that proximal joints may depend more on proprioceptive and vestibular systems than on visual input when processing visual interference. These findings further support the hierarchical contribution theory of multisystem integration in motor control.

## Inter-segment coordination variability (DP)

Consistent with our hypothesis that: the impact of varying degrees of visual differences on lower limb coordination is mainly reflected during specific events for the RHK and RKA coordination (i.e., DSP and RLSP), (Fig. 2), Previous articles have not reported on the inter-segment coordination variability of lateral shuffling, However, past articles have reported that different diseases or intervention methods can make lower coordination variability, such as in gait^29^. Variability, which is a natural and important characteristic of human movement, can be described as the normal variations that occur in motor performance across multiple repetitions of a task^30^. Deviation from these normal variations can lead to biological systems that are overly rigid and robotic, which result in systems that are less adaptable to perturbations, such as those associated with unhealthy pathological states or absence of skillfulness^31^. Our results indicate that the right lower limb, (particularly in hip-knee coordination as the propulsive side, shows heightened sensitivity to visual interference during critical movement phases. Conversely, the left lower limb, serving as the landing support side, demonstrates stronger stability. These findings demonstrate that in lateral movements with a clear functional division, the influence of visual feedback on motor control is closely tied to the limb’s functional role.

The hip-knee coordination variability in the right lower limb (the push-off side) exhibits significant differences between the double support phase (RDSP) and the right leg support phase (RLSP), which may be closely related to the precise requirements for propulsion force and directional control during these phases. During RDSP, the right lower limb needs to generate effective lateral propulsion force, a process that heavily relies on the visual system’s real-time monitoring of spatial position and movement direction^28^. Distorted visual information may impair the central nervous system’s accurate estimation of limb dynamics parameters, thereby reducing the variability of coordination patterns^32^. Especially in RLSP, where the body’s center of gravity is entirely supported by the right lower limb while the left lower limb is at a critical juncture, preparing to enter the swing phase, abnormal visual feedback may further exacerbate the uncertainty in motor execution^32^.

The left lower limb, (serving as the supporting side, maintains stable coordination throughout all phases, which likely reflects the functional priority of the supporting limb in posture control. As the primary weight-bearing limb, the left lower limb plays a critical role in maintaining body stability and balance throughout the entire lateral shuffle step cycle^33^. This functional requirement likely drives the musculoskeletal system to adopt more robust control strategies, prioritizing proprioceptive and vestibular systems over visual feedback to maintain joint coordination^33^, thereby demonstrating relative insensitivity to visual interference. This “stability-first” principle aligns with the optimal allocation strategies of motor control systems in uncertain environments^34^.

Notably, only the variability in coordination between the right knee and ankle showed significant differences at the complete one cycle (ROC) level, whereas hip-knee coordination variability displayed marked variations at specific phases. These hierarchical differences in joint function likely reflect their distinct roles in motor control: the hip joint primarily regulates movement direction and overall strategy, while the knee-ankle joint focuses on execution and fine-tuning. Consequently, visual interference may first affect proximal motor control strategy (manifested in hip-knee coordination) before impacting overall movement patterns (as seen in cyclical coordination)^35^.

As we know, this is the first study to examine RHK, RKA, LHK, and LKA coordination patterns and coordination variability during lateral shuffle in college students. Therefore, direct comparisons of our results with earlier studies are challenging. Additionally, there are various methodologies used to quantify coordination, such as the methods employed by Rei Konishi in 2024^36^. However, this method only employs joint angles as indicators, whereas the present study incorporates both joint angles and angular velocity parameters in the computational approach, which may more comprehensively reveal the various factors influencing coordination.

## Limitations

The limitations of this study are mainly as follows: First, convex lenses were used to manipulate vision in the normal vision group. Although sufficient adaptation time was provided to participants during testing, it may still not fully simulate the myopia state, resulting in certain differences from the characteristics of the myopia Homo sapiens group. Second, the coordination assessment was limited to the sagittal plane. To comprehensively understand the impact of vision on lower limb coordination, future studies should encompass three-dimensional analyses covering the sagittal, coronal, and horizontal planes.

## conclusion

This study systematically investigated the effects of different vision interventions (normal vision, + 150° convex lens, and + 450° convex lens) on lower limb joint coordination during lateral shuffle movement. The main conclusions are as follows: College students with varying degrees of visual impairment demonstrated more in-phase and less variable coordination than those with normal vision in specific lateral shuffle and inter-segmental joint coordination.

Significant changes in visual accuracy substantially impair lower limb motor control, and this interference exhibits clear distal specificity. Research findings indicate that knee-ankle coordination demonstrates greater sensitivity to visual disturbances compared to hip-knee coordination. Particularly under + 450° convex lens conditions, coordination variability decreases significantly (for example, the left knee-ankle coordination showed an η_p_^2^value of 0.418 throughout the cycle).

Consequently, in sports training, for disciplines requiring frequent lateral movements like basketball and badminton, it is recommended to emphasize knee-ankle coordination exercises under visual interference conditions.

## Data Availability

The datasets used and/or analysed during the current study available from the corresponding author on reasonable request.
